# Is a randomised controlled trial of a maternity care intervention for pregnant adolescents possible? An Australian feasibility study

**DOI:** 10.1186/1471-2288-13-138

**Published:** 2013-11-13

**Authors:** Jyai Allen, Helen Stapleton, Sally Tracy, Sue Kildea

**Affiliations:** 1 Midwifery Research Unit, Mater Research, Aubigny Place, Raymond Terrace, South Brisbane, Queensland, Australia; 2 Faculty of Health, School of Nursing, Midwifery and Paramedicine, Australian Catholic University, Nudgee Road, Banyo, Queensland, Australia; 3 Midwifery and Women’s Health Research Unit, University of Sydney, Royal Hospital for Women, Randwick, New South Wales, Australia; 4 Faculty of Medicine, School of Women’s and Children’s Health, University of New South Wales, High Street, Kensington, Sydney, Australia

## Abstract

**Background:**

The way in which maternity care is provided affects perinatal outcomes for pregnant adolescents; including the likelihood of preterm birth. The study purpose was to assess the feasibility of recruiting pregnant adolescents into a randomised controlled trial, in order to inform the design of an adequately powered trial which could test the effect of caseload midwifery on preterm birth for pregnant adolescents.

**Methods:**

We recruited pregnant adolescents into a feasibility study of a prospective, un-blinded, two-arm, randomised controlled trial of caseload midwifery compared to standard care. We recorded and analysed recruitment data in order to provide estimates to be used in the design of a larger study.

**Results:**

The proportion of women aged 15–17 years who were eligible for the study was 34% (n=10), however the proportion who agreed to be randomised was only 11% (n = 1). Barriers to recruitment were restrictive eligibility criteria, unwillingness of hospital staff to assist with recruitment, and unwillingness of pregnant adolescents to have their choice of maternity carer removed through randomisation.

**Conclusions:**

A randomised controlled trial of caseload midwifery care for pregnant adolescents would not be feasible in this setting without modifications to the research protocol. The recruitment plan should maximise opportunities for participation by increasing the upper age limit and enabling women to be recruited at a later gestation. Strategies to engage the support of hospital-employed staff are essential and would require substantial, and ongoing, work. A Zelen method of post-randomisation consent, monetary incentives and ‘peer recruiters’ could also be considered.

## Background

The rising rate of preterm birth (the birth of an infant before 37 completed weeks of pregnancy) is a serious, complex and unresolved public health problem for which there are very few known preventative interventions [1]. Preterm birth is a leading cause of perinatal mortality, serious neonatal morbidity and moderate to severe childhood disability [2-5]. Although preterm births currently comprise 10% of all births internationally [6], they contribute to more than two-thirds of perinatal mortality (fetal loss and neonatal death) [4]. At present there is an incomplete understanding of the mechanisms responsible for spontaneous preterm labour however multiple aetiologies and/or pathological processes are closely associated [3,5].

Idiopathic preterm birth correlates strongly with poverty and lower socio-economic status [7]. Pregnant adolescents are more likely to come from socio-economically disadvantaged backgrounds [8,9]. Maternal age of 17 years or less is considered an independent risk factor for preterm birth [10-14]; whether older teenagers 18–19 years of age are at increased risk of preterm birth is contested [15-17]. The effects of social deprivation on pregnant adolescents are cumulative and multifactorial; they directly affect perinatal outcomes including preterm birth [7]. These include smoking, alcohol and illicit drug use [8,18,19], family violence and/or intimate partner violence [20-22], social isolation [23,24], mental health issues including depression [8,25,26], poor nutrition and inadequate weight gain during pregnancy [25], genito-urinary infection [27,28], and severe psychosocial stressors including low income, unemployment and housing issues [29] or homelessness [30]. These effects are compounded as teenage women tend to book for pregnancy care at a later gestation, attend fewer appointments or attend no antenatal care at all [31,32]. Both non-attendance and under-attendance of antenatal care are independently associated with poor perinatal outcomes including preterm birth [15,32].

Improving adolescent health requires improving the factors that make up young people’s daily lives by addressing the risks and perhaps more importantly strengthening protective factors and resilience [33]. Targeted interventions to address modifiable risk factors for preterm birth have shown promising results, but more research through randomised controlled trial (RCT) design is required [34]. Two models of care demonstrate potential to reduce the preterm birth rate for this population; group antenatal care [34,35] and young women’s clinic [30]. Whether caseload midwifery improves perinatal outcomes for adolescent women has not been tested [36].

The trademarked version of group antenatal care, “Centering Pregnancy”, was designed specifically for socio-economically disadvantaged women including adolescents [37]. In this model groups of 8–12 pregnant women of similar gestation meet regularly for a two-hour facilitated discussion and clinical assessment within the group space [38]. A 2007 RCT of group antenatal care for young women (14–25 years) found it was associated with lower rates of “inadequate prenatal care” (as determined by the Kotelchuck Index [39]), and lower rates of preterm birth [35].

Young Women’s Clinic (YWC) is a model that operates internationally and varies considerably. The key elements include a community clinic setting, multi-disciplinary involvement (including obstetric and allied health presence at the clinic), midwives with additional training, and staff consulting clinical guidelines for working with pregnant adolescents (e.g. sexual health, illicit drug use) [36]. A 2004 prospective cohort study demonstrated that YWC is associated with higher rates of routine antenatal attendance and lower rates of preterm birth (including preterm prelabour rupture of membranes and threatened preterm labor) for women aged less than 18 years [30]. These findings should be interpreted with caution however, given that participants were able to self-select either YWC or standard care [34].

A 2011 systematic review of midwife-led models of care (i.e. team midwifery and caseload midwifery) demonstrated that women who receive this type of maternity care, experience improved maternal and neonatal outcomes without any adverse effects [40]. Caseload midwifery is provided by a small group of midwives who each provide care for a specific caseload of women on an on-call basis; there is an emphasis on providing a known carer in labour with all women having a named midwife [41]. While the systematic review included two RCTs of caseload midwifery; the mean age of participants was 27 years (SD 5 years) in both studies [42,43], hence the findings are not generalisable to the adolescent population. Midwifery group practice (MGP) is a common form of caseload midwifery in Australia (the terms will be used synonymously in this paper) whereby a small group of midwives provide continuity of care throughout pregnancy, birth and the postnatal period for four to six weeks following birth [41]. An Australian multi-centre trial of caseload midwifery, the Midwives at New Group practice Options (M@NGO) trial, was conducted from 2009–2011 [44]. The setting for this feasibility study was one of the sites for the M@NGO trial which included women of ‘all-risk’ status but excluded women aged 17 years or less. The M@NGO trial was not powered to detect a significant difference in preterm birth [44].

We hypothesised that care through a MGP, which incorporates strategies to address the risk factors associated with preterm birth into the one model of care, could decrease preterm birth in pregnant adolescents. We proposed that improving young women’s access to regular, comprehensive antenatal care [35,45-50], and increasing their sense of trust and safety with their midwife [51-53], could affect their willingness to accept infection screening and treatment [30,54], to disclose high-risk behaviors or circumstances [30,55,56], and to adopt strategies which promote health and minimise harm to themselves and their babies [57,58]. Although MGP looked promising as an intervention, we were unsure if pregnant adolescents would agree to be randomised into a study as the literature on pregnant adolescent recruitment is scant; thus a feasibility study was conducted.

## Methods

### Study design and objectives

We have designed an un-blinded, two-arm, randomised controlled trial to analyse Preterm birth Risk for Adolescents in Midwifery group practice or Standard maternity care (PRAMS trial; main study). The primary objective of the PRAMS trial will be to determine whether the proportion of pregnant adolescents experiencing preterm birth less than 37 weeks gestation is similar for those receiving MGP care and those receiving standard care. The current feasibility study was designed to estimate important recruitment parameters needed for the main study, rather than assess the outcome of interest [59,60]. The aim of the feasibility study was to assess the likelihood of recruiting women aged 17 years or less into a RCT of caseload midwifery. The objectives were to test the eligibility criteria, to assess the willingness of potential participants to be randomised and to generate recruitment data to assist in the calculation of the study population required for the PRAMS trial.

### Participants

This feasibility study ran parallel to the M@NGO trial at site two, and recruited women who were ineligible for the M@NGO trial because of their age; otherwise similar eligibility criteria were used [44]. Eligible participants were all women who were 13–17 years of age, who booked for public maternity care at the study hospital, and were 23 weeks pregnant or less, with a single, live fetus at the time of recruitment. Exclusion criteria were maternal age 18 years or older, inability to provide consent (e.g. serious mental illness or lack of English fluency), residence outside of the hospital catchment area (because of the requirement for home visiting), 24 weeks gestation or greater, and multiple pregnancy.

### Ethical aspects

The study received ethical approval from both the Hospital and University Human Research Ethics Committees (HRECs). The Australian National Statement on Ethical Conduct in Human Research recognises that children and young people have different levels of maturity and therefore capacity to make informed decisions about research participation; these levels are not attached to fixed ages [61]. Contemporary Australian law recognises that young women aged 15–17 years may be broadly categorised as “young people who are mature enough to understand and consent, and are not vulnerable through immaturity in ways that warrant additional consent from a parent or guardian” [61]. Women who could not demonstrate that they understood the implications of participation in the study, would have been excluded; however this situation did not occur. Young women aged 13–14 years were considered as competent to understand the relevant information, however their relative immaturity rendered them vulnerable thus, on the advice of the HREC, both participant and parental consent would have been sought; in the event there were no potential participants aged less than 15 years. The Consent Form included that the purpose of the study was to assess the feasibility of conducting a RCT with pregnant adolescents.

### Setting

This Australian-based study took place at an inner-city, tertiary maternity hospital and its associated community-based clinic. The hospital conducts approximately 5000 births for publicly-insured women annually. Women aged 17 years or less account for around 80 births (2%) per annum. The Young Mother’s Partnership Program is an alliance between hospital staff (clinicians and allied health) and a local non-government organisation (NGO) that specialises in supporting pregnant and parenting young women and their families. The NGO provides a community clinic venue with peer support workers who provide assistance with identified needs including housing, income support, health and legal issues, and facilitates access to education and training. Two models of maternity care operate within this setting: MGP for young women (YMGP) and Young Women’s Clinic (YWC); both provide care to women aged 20 years or less. All young women see an obstetrician routinely at 16–18 weeks of pregnancy at the community clinic.

### Intervention and control groups

Women randomised to the intervention (YMGP) received antenatal, intrapartum and postnatal care from a known midwife. Women randomised to the control group were able to select any other available model of antenatal care including YWC, care with a general practitioner, or a community or hospital-based antenatal clinic. For a detailed description of the differences between YMGP and other models of maternity care, see Table 1.

**Table 1:** Differences between YMGP and other types of maternity care

	**Intervention**	**Control**
**Model of maternity care**	Young women’s Midwifery Group Practice (YMGP)	Young Women’s Clinic (YWC)
		GP shared care
		Antenatal clinic in the hospital or community outreach clinics
**Booking appointment**	YMGP midwife conducts a home visit	Rostered midwife conducts the booking visit in hospital or a community outreach clinic
**Antenatal care**	YMGP midwives provide group antenatal care in the community venue.	Rostered midwife or doctor in the hospital antenatal clinic or community outreach clinics
	Individual visits with the obstetrician or social worker at the community venue as part of routine care	Referral to social worker if indicated
**Antenatal education**	Education is incorporated into the group antenatal care sessions; no separate classes	YWC clients can access specific ‘active birth’ classes for young women at community venue
		Young women in all other models of care can access standard classes at the hospital
**After hours contact**	YMGP midwife via mobile telephone; diverted to a back-up YMGP midwife when required	Rostered midwife via hospital telephone number
**Intrapartum care**	YMGP midwife in the hospital assessment unit or birth suite	Rostered midwife or doctor in the hospital assessment unit or birth suite
**Inpatient postnatal care**	Rostered midwife or doctor in the public postnatal ward	
**Outpatient postnatal care**	YMGP midwife home visits for 4–6 weeks following birth	Rostered midwives provide two to three home visits until 10–14 days after birth for women in the hospital catchment area
	Young women invited to the community clinic venue for a Postnatal Group	

### Outcomes

The primary outcome measure for the PRAMS trial will be the proportion of women who experience preterm birth. The secondary outcome measures will include gestation, birth weight, mode of birth, Apgar score less than 7 at 5 minutes, breastfeeding initiation and at hospital discharge, admission to a separate neonatal nursery, length of maternal and neonatal stay.

The feasibility study outcomes included the proportion of participants who were eligible, willing to be randomised, withdrew from the study, were lost to follow up, and changed model of care (cross-over). The criteria for determining feasibility were eligibility and recruitment rates of 65% or more; which were based on rates achieved in the RCT of group antenatal care with young women [35]. Simple descriptive statistics were used to analyse the feasibility outcomes.

### Sample size

For the PRAMS trial we calculate we would have 80% power to detect a 33% reduction in preterm birth (p < 0.05) with a targeted sample size of 1864 (n=932 in each group). A feasibility study is not powered to detect a statistically significant difference on any measure. With a six month recruitment period we estimated we could assess approximately 40 pregnant adolescents for eligibility and that 20 participants could be recruited.

## Results

### Recruitment

**Figure 1:**
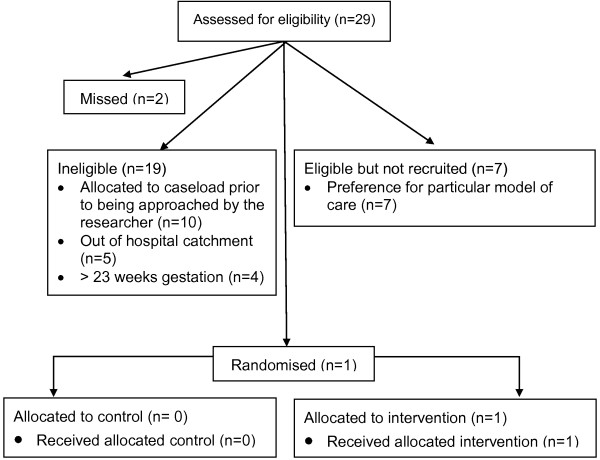
Participant flow diagram.

Recruitment occurred during October 2010 to March 2011. The flow of participants through each stage of recruitment is described in Figure 1. The GP referral letter to the hospital was used to identify women who were eligible to participate. Eligible women received the routine letter of hospital acceptance and a brochure describing models of maternity care; with the addition of a M@NGO trial brochure. Telephone recruitment was the initial method used to approach participants.

### Telephone recruitment

Approximately five days after the research information was posted to potential participants, the research midwife attempted to telephone them on three separate occasions. The telephone call aimed to ensure the study information was received, confirm eligibility, answer any questions, and offer participation in the study. If the woman gave verbal consent to participate, she was randomised to intervention or control using a random sequence of envelopes. Participant details were then entered on the research database. Women allocated to the intervention (YMGP) confirmed and formalised participation in the trial by giving written consent at the first booking visit (in the home). In the control group, written consent was obtained at the first booking visit in the hospital or community-based antenatal clinic. Women who refused to give written consent were excluded from the trial. Less than 20% (n = 5) were contactable by the telephone method described above. If women were un-contactable after three separate attempts, then the referral was returned to the administration office to make a first booking appointment with the YWC midwife. The researcher undertook a parallel process of face-to-face recruitment at the community clinic. This recruitment process described is similar to the method conducted successfully in the M@NGO trial [44].

### Face-to-face recruitment

Most potential participants (n = 22) were approached by the research midwife at their routine obstetric visit. Women were given an opportunity to discuss the study and review the Participant Information Sheet and Consent Form. If written consent was obtained, women were randomised as per protocol. If women declined to participate, or were found to be ineligible, their reason for declining or ineligibility was recorded on the research database.

### Outcomes

Twenty-nine young women aged 15–17 years were assessed for eligibility, 66% (n = 19) were deemed ineligible because they were: already booked into YMGP (n = 10), out of the hospital catchment area (n = 5), or more than 23 weeks gestation (n = 4). This resulted in only a small pool of eligible women (n = 10) of which 70% (n = 7) declined to be randomised, 20% (n = 2) were missed, and 10% (n = 1) were recruited; see Figure 1. All the eligible participants who declined to be randomised expressed a strong preference for a particular model of care: YMGP (n = 4), GP shared care (n = 2), or the antenatal clinic (n = 1). Two women were missed by the researcher because they were un-contactable by telephone and repeatedly did not attend their obstetric booking appointment. Only one young woman was able to be recruited; therefore the proportion of women withdrawing, being lost to follow up, or crossing over from one model to the other was unable to be calculated due to the small sample size.

## Discussion

Difficulty recruiting pregnant adolescents does not justify their exclusion from research [62], particularly when they are at higher risk of adverse perinatal outcomes including preterm birth. Health research with low-income pregnant participants suggests it is ideal to access a study population at least double the size of the intended sample [62]. For the PRAMS trial this would mean access to approximately 4000 pregnant adolescents. If the research protocol was used without modification and 4000 young women were screened for eligibility, approximately 1360 would meet the eligibility criteria (34%) and of those around 136 would be recruited (10%). This is clearly not feasible. An effective research protocol could be developed through modification of the eligibility criteria, recruitment strategies, and research design.

### Eligibility criteria

The age of participants was limited to women 17 years or less, which typically accounts for a very small proportion of the pregnant population, including the adolescent pregnant population. This age limit was chosen because the feasibility study ran alongside the M@NGO trial, which included women 18 years and older and we did not want to threaten recruitment to M@NGO in any way. Including participants aged 19 years or less would double the pool of potentially eligible pregnant adolescents at this site.

A small proportion of women (14%, n = 4) were ineligible to participate because they were 24 weeks gestation or greater by the time they were approached about the research. Pregnant adolescents often book late for pregnancy care [32] and are more likely to be un-contactable by traditional methods for research follow up [45]. Therefore including participants at a gestation of 27 weeks or less would be useful to capture those women who book later for antenatal care. This would balance a gestation cut-off that is early enough to give participants time to be exposed to the intervention (YMGP) prior to birth. This is particularly significant for the PRAMS trial, as the primary outcome will be preterm birth (< 37 weeks gestation).

Four of the five most disadvantaged areas are outside the catchment area of this inner city hospital [63]. During the time of the trial women considered disadvantaged (e.g. young or Aboriginal and Torres Strait Islander women) were accepted to the hospital for maternity care if they resided on the south-side of the city even if it was outside the hospital catchment area. However access to YMGP, with associated home visiting, was strictly limited to women in the hospital catchment area. This meant that 17% of young women (n = 5), who lived outside the designated area, were ineligible to participate. Flexibility regarding the hospital catchment area would increase the pool of the eligible young women in this study setting.

### Recruitment plan

Telephone contact was chosen as a recruitment method for two reasons. Firstly, women allocated to YMGP receive their first booking visit in the home, and this is considered an important element of the intervention. Therefore randomisation ideally needed to occur prior to the first booking visit. Secondly, this method is effective in recruiting potential participants who do not respond to a written research invitation [64]. This was effective at our site in recruiting 18–21 year old women into the M@NGO trial; more than 70% had been contactable by telephone and approximately 50% were recruited over the telephone. Nevertheless, most women in the feasibility study were not contactable because mobile telephone numbers were not provided or were disconnected, or telephones were switched off, or telephone calls were simply not answered. Anecdotally, it is not uncommon for people to leave telephone calls unanswered when the telephone number displays as unknown or ‘blocked’ (which it does from any hospital extension number). When a similar problem was encountered in the M@NGO trial, the research midwives made contact through text message in the first instance and invited the women to telephone the researcher at the hospital. This successful strategy was not repeated here, due to ethical considerations and limited time resources. Ethical considerations included the potential to cause harm by leaving a text message that unintentionally alluded to an undisclosed adolescent pregnancy. While a fully-funded RCT, like the M@NGO trial, enabled a researcher to recruit every weekday, this unfunded feasibility study allowed one day per week for recruitment. Therefore the researcher was not able to reliably or promptly answer the phone calls of potential participants who may respond to a text message. Nevertheless, the use of text message to follow up potential research participants, after a research pack has been posted, could be considered as a modification for the PRAMS trial.

Prior to recruitment there was one meeting between the YMGP midwives, their manager, the lead obstetrician and the research team. The researcher then discussed study recruitment with the YMGP midwives at the community clinic on a weekly basis. The YMGP midwives consistently expressed concerns that involvement in the study would result in young women being randomised out of their service. This was troubling to the midwives because they strongly believed that caseload care was the most appropriate model for pregnant adolescents. A 2002 Australian study of paediatric support for RCTs involving children, found that those clinicians with research experience were the most supportive, while those with a strong preference for a particular intervention hindered recruitment [65]. Thus, it is understandable that the YMGP midwives, who perhaps had little experience of research themselves and a strong personal investment in the intervention (YMGP), would not support the feasibility RCT. Furthermore, the midwives voiced concerns that if women were randomised to other models of care, then they would fail to meet their minimum caseload requirements, with imagined implications from management. Despite official management approval for the trial, there seemed to be lingering budgetary and job security concerns if the YMGP was not operating at full capacity. The notion that half of all women who met YMGP criteria and were willing to accept YMGP care would be randomised elsewhere, was understandably troubling to all staff who are invested in demonstrating a sustainable service. In this context, it is perhaps unsurprising that the YMGP midwives booked 34% (n = 10) potential participants into caseload midwifery care prior to the women being approached about the research.

Two other strategies that have demonstrated effectiveness in recruiting adolescents and/or women from minority groups to a RCT could be considered as protocol modifications. First, offering incentives to adolescents who complete postal research questionnaires is known to be an effective strategy, therefore monetary incentives to promote adolescent participation in a RCT could be considered [66]. A second strategy would be to train ‘peer recruiters' , other young women already enrolled in the trial, to disseminate information and discuss the research with other pregnant adolescents at the community venue. This method has showed promising results for increasing recruitment rates of other minority groups (i.e. Hispanic women in the United States) [66].

### The randomised controlled trial design

Once young women were informed of their options for maternity care, the majority of eligible participants declined participation in the study so that they could choose their preferred model of care. The mothers of pregnant adolescents often play an important role in their daughter’s decision-making processes [67], and some mothers voiced concerns about maternity care that was not provided by a doctor. Some young women didn’t actually decline but rather became confused by being presented with options for care that were then removed by randomisation. In addition to being young, pregnant adolescents tend to live with circumstances of socio-economic deprivation including poor educational opportunities and achievements [8,68]. Perhaps it is not surprising then, that the concept of randomisation for research purposes was difficult to understand and this became a barrier to participation in the study. A Zelen randomised consent design where eligible participants are randomly allocated to the intervention or control group prior to being approached about the trial or gaining consent should be considered. Those participants allocated to the intervention group are then approached and offered the intervention, which they can decline or accept; those allocated to the control group are also approached to participate [69]. This design has been used successfully in other trials of maternity care interventions [70-72], including those with pregnant adolescents [73]. Giving participants the opportunity to ‘opt out’ of the research, rather than ‘opt in' , has been shown to increase participation in survey research [74]; and could assist in increasing recruitment for the PRAMS trial.

## Conclusions

Our study demonstrated that an RCT of caseload midwifery which exclusively recruited pregnant adolescents (aged 17 years or less) using the eligibility criteria, recruitment plan and post-consent randomisation method tested would not be feasible without modification. Eligibility criteria which include adolescent participants up to 19 years of age, with a gestation of up to 27 weeks, and more relaxed catchment boundaries, could increase the pool of eligible women. It would be useful to consider a Zelen method of post-randomisation consent where participants need to ‘opt out’ of the study, monetary incentives for participation, and employing ‘peer recruiters’ to address the recruitment barriers described.

## Competing interests

The authors declare that they have no competing interests.

## Authors’ contributions

JA participated in the design of the study, conducted recruitment, interpreted data, and drafted the manuscript. HS assisted with data interpretation and has been involved in critical revisions of the manuscript. ST was primarily responsible for the conception and design of the M@NGO trial on which this feasibility study was based; she has been involved in critical revisions of the manuscript. SK participated in the design of the study, and has been involved in critical revisions of the manuscript. All authors read and approved the final manuscript.

## Pre-publication history

The pre-publication history for this paper can be accessed here:

http://www.biomedcentral.com/1471-2288/13/138/prepub
